# Salivary and lacrimal disorders in patients treated with radioiodine for differentiated thyroid cancer

**DOI:** 10.1530/ETJ-25-0402

**Published:** 2026-05-18

**Authors:** Soline Bondet de La Bernardie, Marie-Odile Bernier, Camille Buffet, Charlotte Lussey-Lepoutre, Clémence Baudin

**Affiliations:** ^1^ Autorité de Sûreté Nucléaire et de Radioprotection (ASNR), PSE-SANTE/SESANE/LEPID, Fontenay-aux-Roses, France; ^2^ Thyroid and Endocrine Tumors Department, Pitié-Salpêtrière Hospital, Thyroid Tumors Clinical Research Group n°16, Sorbonne University, Cancer Institute, Inserm U1146, CNRS UMR, Paris, France; ^3^ Sorbonne University, Unit of Radionuclide Treatment, Nuclear Medicine Department, Groupe de Recherche Clinique Tumeurs Thyroïdiennes n°16, AP-HP, Pitié-Salpêtrière Hospital, Paris, France; ^4^ Centre de Recherche des Cordeliers, INSERM U1138, Equipe Labellisée par la Ligue contre le Cancer, Paris, France

**Keywords:** thyroid, salivary dysfunction, xerostomia, lacrimal, I^131^

## Abstract

Radioactive iodine therapy (RIT) is commonly used as an adjuvant treatment for differentiated thyroid cancer (DTC). While its efficacy is well established, this therapy can cause radiation-induced damage to the salivary and lacrimal glands due to their ability to concentrate iodine. These adverse effects may lead to xerostomia, sialadenitis, or xerophthalmia. The aim of this review and meta-analysis was to estimate the prevalence and characteristics of salivary and lacrimal dysfunctions in patients treated with RIT for DTC. Following PRISMA guidelines, a systematic search was conducted in PubMed and Scopus databases to identify studies published between 2000 and 2024. Meta-analyses of prevalence and odds ratios were carried out using random-effects models to estimate the pooled prevalence of events and the influence of administered activity. Thirty-eight studies were included in the review, of which sixteen were retained for the meta-analyses. The pooled prevalence of xerostomia decreased from 60% (95% CI: 0.28–0.85) at <2 months to 17% (95% CI: 0.10–0.28) at more than 1 year post-RIT. A significant association was observed between high levels – with study-specific thresholds – of administered I^131^ activity and the occurrence of xerostomia. Sialadenitis and lacrimal dysfunctions were also frequently reported. Despite heterogeneity in protocols and assessment tools, most studies highlighted a frequently persistent impairment in many patients. This systematic review and meta-analysis highlighted the frequency and persistence of salivary and lacrimal dysfunctions following RIT.

## Introduction

Differentiated thyroid cancer (DTC) generally has a favorable prognosis, with a 10-year survival rate of over 90% for papillary thyroid carcinoma, the most common histological type. Its incidence has been rising in France, since 1980s, with an estimated average annual growth rate of 4.4% between 1990 and 2018 (1). In 2018, there were more than 10,000 new cases, three quarters of which involved women (1). The usual treatment of DTC consists of surgery with partial or complete thyroidectomy, completed usually by radioiodine (I^131^) therapy (RIT) administered to the patient (2). However, the usefulness of the latter is debated especially in low-risk cancer due to the possibility of short-, medium-, or long-term side effects (3). Moreover, several studies have shown that RIT does not significantly improve either overall survival or recurrence rates in patients with low-risk DTC (4, 5). Indeed, salivary glands can concentrate iodine, which can cause significant inflammation when administering I^131^; this inflammation is likely to lead to chronic salivary complications that can cause deterioration in oral health (6). One of the most common side effects reported by patients after treatment is a dry mouth sensation (xerostomia), caused by a damage to the salivary glands (7). Similarly, the lacrimal glands are also capable of trapping I^131^, leading to radiation-induced injury as dry eyes (xerophthalmia), or eye discomfort, which may appear months after treatment (8). Some factors can influence the severity of these symptoms, such as age, sex, or the method used to stimulate the remaining thyroid cells (9, 10, 11, 12). Thus, the objective of this review is to examine the existing literature on salivary and lachrymal disorders in patients treated with I^131^ for thyroid cancer, by identifying relevant studies and providing extensive analysis and quantitative summary of these adverse effects in the short, medium, and long term.

## Materials and methods

This systematic review was conducted following the Preferred Reporting Items for Systematic Reviews and Meta-Analyses (PRISMA) guidelines. The review protocol was registered in the international systematic review registry PROSPERO (registration number: CRD42024625282).

### Eligibility criteria

Eligible studies included those conducted on thyroid cancer patients treated with I^131^. Observational studies, including cohort, longitudinal, case control, and cross-sectional designs, were considered for inclusion. Abstract articles only, books, editorial, theses, and case reports were excluded. The review was restricted to studies published in English between January 2000 and December 2024.

### Sources and search strategy

The bibliographic databases Scopus and PubMed were searched using combinations of the following terms: ‘thyroid’, ‘cancer’, ‘papillary’, ‘follicular’, ‘salivary dysfunction’, ‘salivary glands’, ‘eye dryness’, ‘mouth dryness’, ‘oral dryness’, ‘xerostomia’, ‘hyposalivation’, ‘sialadenitis’, ‘saliva’, ‘radioiodine’, ‘I131’, ‘radioactive iodine’, and ‘radiation therapy’. Studies containing the keywords ‘animal’, ‘cat’, ‘hypothyroidism’, or ‘Graves’ disease’ were not retained in the search. Title extraction was performed on January 13, 2025.

### Study selection and data extraction

The selection process was carried out in several stages. Titles identified from the database search were first screened independently by two investigators (CB and SB). Abstracts of the selected titles were then reviewed by the same investigators. The independent selections from the two investigators were compared, and in case of disagreement, a third investigator (M-OB) made the final decision. The last selection was carried out with the full-text reading by one reviewer (SB) and the followings information were saved in a table: title, authors, country, date of publishing, type of study, outcome, characteristics of study population, inclusion criteria, measuring tools, administered activity, following points information and duration of follow-up, results, conclusions, advantages, and limits of the study in the author’s discussion.

### Study bias assessment

The quality of included studies was assessed using the Newcastle–Ottawa Scale (NOS). The assessment focused on three domains: quality of selection, comparability of study groups (based on design or analysis controlling for confounders), and the assessment of outcomes.

### Statistical analysis

Meta-analyses were carried out when at least three studies for a studied outcome were available. Meta-analyses of prevalence were performed to estimate the combined prevalence of adverse events (xerostomia and sialadenitis). Analyses were conducted using a random effects model, by applying the inverse variance method, where each study is weighted according to the precision of its estimator (13). Inter-study variance (*τ*^2^) was estimated using DerSimonian–Laird method.

Another meta-analysis was conducted to assess the risk of xerostomia in relation to the I^131^ activity administered. This analysis used a DerSimonian–Laird random effects model applied to odds ratios (OR). Egger’s tests were performed for each meta-analysis to assess the presence of publication bias, and the corresponding results are provided in the supplementary material (Figs S2, S3, and S4 (see section on Supplementary materials given at the end of the article)). The results of the meta-analyses were presented in forest plots, illustrating individual and combined estimates with their 95% confidence intervals. Statistical significance was set at a threshold of *P* < 0.05. All statistical analyses were performed using R 4.5.0 software (R Foundation for Statistical Computing, Austria), using the meta and metafor packages.

## Results

### Identification of the included studies

From the 3,825 records initially identified through Scopus and PubMed searches, 589 duplicates were excluded, leaving 3,236 records for title screening. Of these, 89 were selected for abstract review. Along with 3 additional records from other sources, 57 were deemed eligible for full-text assessment.

After reviewing the 57 full texts, 19 studies were excluded for the following reasons: 5 due to study design, 7 due to characteristics of the study population, 3 because the full text was not accessible, 3 due to publication date, and 1 due to the type of exposure assessed (including chemotherapy). Ultimately, 38 studies published between 2000 and 2024 were included in this review, 16 of which were used for meta-analyses. Regarding temporal distribution, 2 studies were published between 2000 and 2005, 5 between 2006 and 2010, 7 between 2011 and 2015, 17 between 2016 and 2020, and 7 between 2021 and 2024. Most of the included studies were observational, especially cohort (26 studies) and cross-sectional (9 studies) (Fig. S1).

Most studies reported results on the most common symptoms, such as xerostomia (24 studies), glandular swelling and pain (16 studies), or sialadenitis (8 studies). Some other effects were also reported, though less frequently, including xerophthalmia (5 studies) or dysphagia (3 studies).

### Short-term effects (<2 months)

Nine articles assessed adverse events occurring within 2 months after RIT (Supplementary Tables 1 and 2). Eiras Ramim *et al.* (14) and Ming *et al.* (15) reported a significant increase in xerostomia symptoms within the first week following RIT like Goswami *et al.* (16), who described a xerostomia rate reaching 73% (mild to severe) within a few days to weeks after treatment. In the pediatric cohort studied by Albano *et al.* (17), 14% of patients reported dry mouth less than 1 week after RIT. Lopes da Fonseca *et al.* (18) and Tabari *et al.* (19) observed a significant decrease in salivary flow. Daniel *et al.* (20), however, reported no significant change in salivary flow but found a significant reduction in calcium and phosphate salivary concentrations, as well as an increase in dysphagia (20). The prevalence of sialadenitis was reported in several studies, with rates of 58% (mild to severe) reported by Goswami *et al.* (16) and 22% among children reported by Albano *et al.* (17). Similarly, Hyer *et al.* (21) observed salivary toxicity in 26% of patients, most commonly presenting with pain and swelling. Badam *et al.* (22) demonstrated a significant reduction in salivary uptake using 99mTc-pertechnetate and in ejection fraction following RIT.

### Mid-term effects (2–6 months)

Six articles assessed adverse events occurring between 2 and 6 months after RIT (Supplementary Tables 1 and 2). Bulut *et al.* (23) and Klein Hesselink *et al.* (24) reported a significant increase in xerostomia at 3 and 5 months, respectively, after RIT compared to baseline, with rates rising from 4 to 20%. Hyer *et al.* (21) observed acute toxicity in 12% of patients, including pain, swelling, and xerostomia 3 months post-RIT, like Eiras Ramim *et al.* (14) who reported persistent mouth and throat pain, as well as swallowing difficulties. Klein Hesselink *et al.* (24) and Lopes da Fonseca *et al.* (18) observed a significant decrease in stimulated salivary flow at 5 months and at 2 and 4 months, respectively, and in unstimulated flow at 5 and 4 months, respectively. Conversely, Krčálová *et al.* (25) found no significant differences in salivary gland uptake or excretion fraction before and after treatment at 4–6 months post-RIT.

### Mid- and long-term effects (6–12 months)

Four articles assessed adverse events occurring between 6 and 12 months after RIT (Supplementary Tables 1, 2, and 3). Lee *et al.* (26) reported that 21% of patients developed symptoms within 6–12 months following RIT, including xerostomia, swelling, and pain. Baudin *et al.* (27) confirmed the onset of new symptoms, with 22% of patients reporting xerostomia. Lopes da Fonseca *et al.* (18) and Baudin *et al.* (27) observed a significant decrease in stimulated salivary flow at 6 months, accompanied by an increase in potassium concentration but no change in unstimulated salivary flow (27). Nabaa *et al.* (28) observed a reduction in the volume of the parotid and submandibular glands, correlated with increasing dysfunction grade, and a significant increase in parotid gland attenuation on CT according to a dysfunction grad, though no such change was observed for the submandibular gland.

### Long-term effects (>12 months)

Twelve studies have reported adverse effects persisting for more than 1 year after RIT (Supplementary Tables 1, 2, and 3). Xerostomia remains the most frequently observed symptom, with incidences ranging from 15% at 1 year (29) to 44% after a median follow-up of 6.6 years (30). Intermediate rates have also been reported: 17% (31), 31% (32), and 33% (33, 34), with the prevalence gradually decreasing but remaining at 15% after 3 years (34). Xerostomia was further observed in 16% of patients at 5 and 14–17 years post-RIT (35, 36) and in 36% of children 11 years after treatment (37). Sialadenitis is also a frequent complication, reported in 22–24% of cases according to Missaoui *et al.* (38) and Walter *et al.* (30) after approximately 6 years of follow-up. Hyer *et al.* (21) and Horvath *et al.* (9) described chronic toxicity characterized by pain – particularly in the parotid glands – accompanied by taste alterations (9) and dysphagia (9, 35).

### Side effects related to the I^131^ activity

Several studies have highlighted a relationship between RIT-delivered activity and xerostomia symptoms (Supplementary Tables 1, 2, and 3). Baudin *et al.* (27) reported an OR of 1.43 per Gy (95% CI: 1.02–2.04); Walter *et al.* (30) reported an OR of 1.15 (95% CI: 1.04–1.29) per GBq; and Hollingsworth *et al.* (39) reported an OR of 4.01 (95% CI: 1.81–8.90) for patients who received RIT compared with those who did not. Baudin *et al.* (27), Klein Hesselink *et al.* (24), and Almeida *et al.* (40) observed a reduction in stimulated salivary flow rate associated with cumulative activity. Selvakumar *et al.* (37) and Badam *et al.* (22) found a significant decrease in uptake and ejection fraction related to administered activity. Dingle *et al.* (41) and Hollingsworth *et al.* (39) found a significant association between administered activity and sialadenitis, with HR = 7.43 (95% CI: 1.67–33.01) for patients who received RIT compared with those who did not, respectively. Makarenko *et al.* (42) reported a 51% rate of chronic sialadenitis. Solans *et al.* (34), Lee *et al.* (26), Caglar *et al.* (43), and Wu Feng *et al.* (44) reported an increased incidence of salivary gland dysfunction with the number of treatments and cumulative administered activity, particularly beyond 22.2 GBq. Florenzano *et al.* (12) observed symptoms at doses as low as ≤1.85 GBq, while Huang *et al.* (33) and Almeida *et al.* (45) reported a markedly reduced risk of salivary gland dysfunction for patients receiving a cumulative ^131^I activity ≤5.55 GBq compared with those receiving >12.95 GBq (OR = 0.063; *P* < 0.001) (33). Horvath *et al.* (9) and Selvakumar *et al.* (37) found greater gland impairment from 7.4 GBq (OR = 1.32, 95% CI: 1.09–1.61 per GBq). Similarly, Jeong *et al.* (36) and Grewal *et al.* (46) observed a positive correlation between salivary gland dysfunction and administered activities in the category 2.78–5.55 GBq. In contrast, other studies did not find a significant association. Almeida *et al.* (40) reported no relationship between xerostomia and RIT (*P* = 0.63), nor between stimulated and unstimulated salivary flow (*P* = 0.17 and *P* = 0.10, respectively). Geres *et al.* (47) also found no evidence of a cumulative I^131^ effect on sialadenitis, consistent with the findings of Walter *et al.* (30) (OR = 1.03; *P* = 0.53). Krčálová *et al.* (25) reported no significant alteration of the salivary glands after administered activity of 3.7 GBq. Similarly, Chow *et al.* (31) found no significant difference in dysphagia (*P* = 0.27) or dysphonia (*P* = 0.44) between treated and untreated patients. Finally, Iakovou *et al.* (48) observed no correlation between administered activity and salivary gland dysfunction, with rhTSH appearing to reduce toxicity.

### Lacrimal adverse effects

Within 2 months after RIT, Goswami *et al.* (16) reported xerophthalmia in 45% of patients, a symptom similarly observed by Lopes da Fonseca *et al.* (18) 2 months after RIT. According to Lee *et al.* (26), 29% of symptoms appeared within 1 week following RIT. Baudin *et al.* reported 17% of patients reporting xerophthalmia at 6 months (27). Xerophthalmia was also reported by Solans *et al.* (34) (25%) and Horvath *et al.* (9) (15%) at 1 year. Hedman *et al.* (35) and Grewal *et al.* (46) documented persistent symptoms in 4 and 5% of patients at 14 and 7 years after RIT, respectively, whereas Lee *et al.* (26) observed that approximately 13% of patients developed new symptoms after 1 year (Supplementary Tables 1 and 3).

### Results of meta-analysis

#### Xerostomia

**Figure 1:**
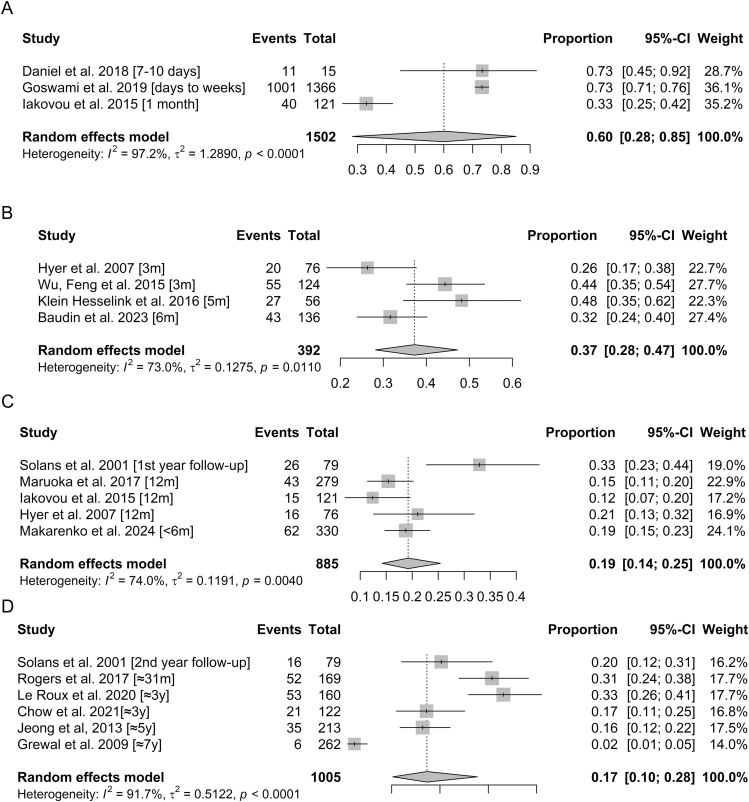
Forest plots for meta-proportions of xerostomia at different times post-RIT: within 2 months (A), between 2 and 6 months (B), between 6 months and 1 year (C), and beyond 1 year (D).

The pooled prevalence of xerostomia was estimated at 60% (95% CI: 0.28–0.85) within 2 months following RIT, based on a random-effects model including 3 studies with a total of 1,502 patients, showing very high heterogeneity (*I*^2^ = 97.2%, *P* < 0.0001) (Fig. 1A). Between 2 and 6 months after treatment, this prevalence was estimated at 37% (95% CI: 0.28–0.47), based on four studies including a total of 392 patients, with substantial between-study variability (*I*^2^ = 73.0%, *P* = 0.0110) (Fig. 1B). For the 6–12-month period, the pooled prevalence reached 19% (95% CI: 0.14–0.25), calculated from 5 studies for a total of 885 patients, still showing high heterogeneity (*I*^2^ = 74.0%, *P* = 0.0040) (Fig. 1C). Finally, more than 1 year after RIT, the estimated prevalence of xerostomia was 17% (95% CI: 0.10–0.28), according to 6 studies with a total of 1,005 patients, showing very high heterogeneity (*I*^2^ = 91.7%, *P* < 0.0001) (Fig. 1D).

**Figure 2:**
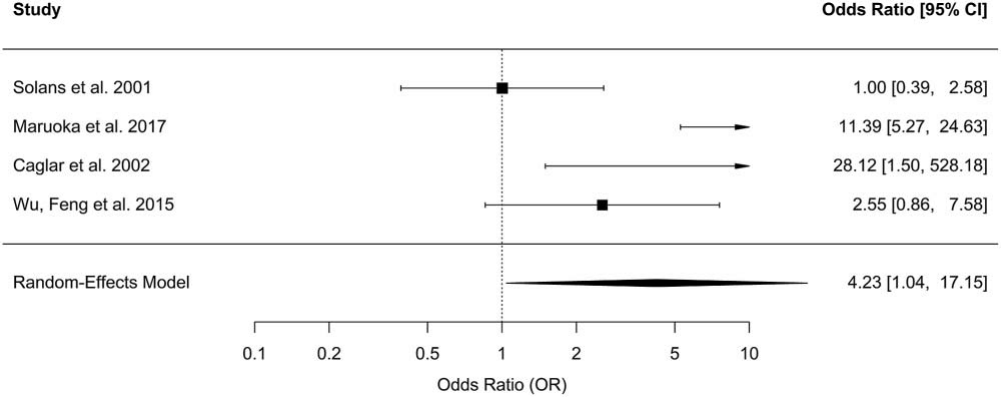
Forest plot for the meta-OR of xerostomia comparing low vs high activity administration.

Then, a pooled OR was calculated from four studies, comparing the risk of xerostomia for different I^131^ activity groups. The study by Solans *et al.* (34) compared ≤3.7 GBq vs ≥7.4 GBq; Maruoka *et al.* (29) compared ≤5.07 GBq vs >5.07 GBq; Caglar *et al.* (43) compared ≤5.55 GBq vs ≥7.4 GBq; and Feng *et al.* (44) compared ≤5.55 GBq vs >5.55 GBq. The meta-analysis showed an overall increased risk of xerostomia in patients whose I^131^ activities were considered high compared with those whose activities were considered low, respectively in each study (OR = 4.23; 95% CI: 1.04–17.15) (Fig. 2). The confidence intervals among individual studies were wide, indicating variability in the precision of estimates. A sensitivity analysis was conducted excluding the study by Solans, whose ‘low’ activity threshold differed the most from that of the other included studies. After exclusion, pooling of the three remaining studies resulted in an estimated OR of 7.14 (95% CI: 2.02–25.26).

#### Sialadenitis

**Figure 3:**
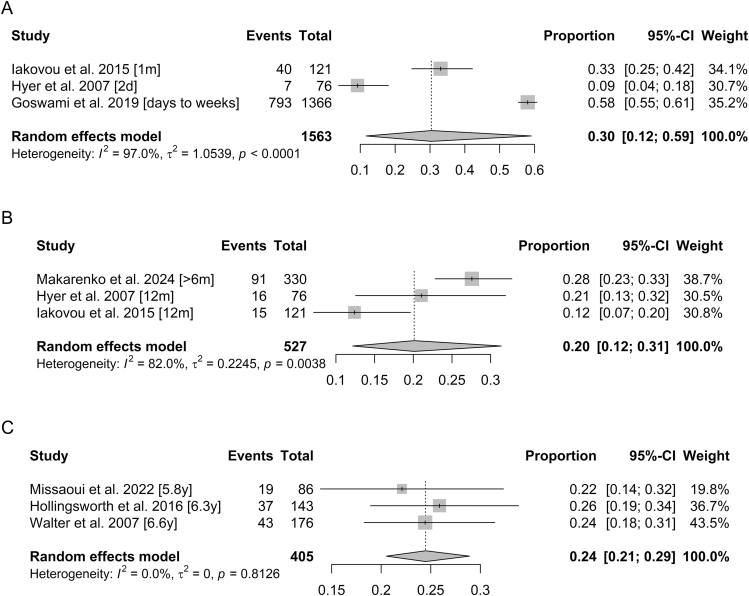
Forest plots for meta-proportions of sialadenitis within 2 months (A), between 6 months and 1 year (B), and beyond 1 year (C).

The pooled prevalence of sialadenitis was estimated at 30% (95% CI: 0.12–0.59) within 2 months following RIT, including three studies and a total of 1,563 patients (Fig. 3A). Inter-study variability was very high, with substantial heterogeneity (*I*^2^ = 97.0%, *P* < 0.0001). Too few studies assessed sialadenitis between 2 and 6 months after RIT to allow for a meta-analysis during that period. Between 6- and 12-month post-treatment, the estimated prevalence was 20% (95% CI: 0.12–0.31), based on three studies including a total of 527 patients, again with important heterogeneity (*I*^2^ = 82.0%, *P* = 0.0038) (Fig. 3B). Beyond 1 year, the estimated prevalence was 24% (95% CI: 0.21–0.29), derived from three studies involving a total of 405 patients, with no observed heterogeneity (*I*^2^ = 0%, *P* = 0.8126) (Fig. 3C).

## Discussion

Based on the 38 studies which fulfilled the inclusion criteria, our review showed that the most frequently reported symptoms after RIT may last for several months or years regarding xerostomia, sialadenitis, pain, and swelling (21, 22, 28, 30, 31, 41, 49); but these do not indicate any major clinical disorders. The results from 16 studies on different salivary gland dysfunction were combined using meta-analyses confirming that such disorders often appear within the first few weeks after RIT and may persist for several months or even years but seem to decrease over time (Fig. 1). The wide confidence intervals observed in our pooled estimates reflect a substantial degree of statistical uncertainty. Consequently, these wide intervals should be interpreted as an indication of the need for larger, prospective, and methodologically standardized studies in order to provide more precise and reliable estimates of the true prevalence of salivary toxicity after RIT. The results of this meta-analysis should, therefore, be interpreted with caution due to the substantial methodological and clinical heterogeneity among the included studies. Differences in the populations studied, the definition and categorization of activity levels, measurement tools, and follow-up durations limit the direct comparability of estimates. The use of a random-effects model allows for the incorporation of this variability, but it does not enable the identification of its underlying determinants. Consequently, the pooled odds ratio should be considered as an indication of general trends rather than a precise estimate applicable to a specific exposure threshold. Although subgroup analyses or meta-regression could theoretically help identify potential sources of heterogeneity, such analyses were not feasible in the present study. The number of studies available for each outcome and time interval was limited, and several strata included fewer studies than generally recommended for reliable meta-regression. We did not include studies focusing only on very short-term disorders occurring within the first 2 days after treatment by radioiodine, as reported by Pak *et al.* and Schlumberger *et al.* (50, 51). Indeed, these effects – being mostly transient – do not appear to have a lasting impact on patients’ quality of life. Regarding the included studies, several have examined the dose–response relationship between the administered activity and adverse effects. Our meta-analysis confirmed an association between high levels – as defined in the individual studies (≤3.7 GBq vs ≥7.4 GBq, ≤5.07 GBq vs >5.07 GBq, and ≤5.55 GBq vs ≥7.4 GBq or ≤5.55 GBq vs >5.55 GBq) – of administered I^131^ activity and xerostomia (Fig. 2). However, it is noteworthy that it has been difficult to define a common activity threshold above which the risk increases significantly, due to the heterogeneity of exposure categories across studies – with different definitions of activity ranges, cumulative doses, and group stratifications – which prevents direct and consistent comparisons. Sensitivity analysis was conducted for the meta-analysis of OR for xerostomia comparing ‘low vs high’ activity, excluding the study by Solans *et al.* due to its markedly different definition of the ‘low’ activity threshold compared with the other studies included in the meta-analysis. Although heterogeneity between studies remained high, the overall estimates maintained the same trend. The pooled odds ratio comparing ‘low’ vs ‘high’ administered activity should be interpreted with caution. What is considered ‘high’ in one study may be classified as ‘low’ in another. Therefore, the overall OR reflects only a general trend toward an increased risk of xerostomia with higher activity levels, rather than the effect of a specific threshold. This variability limits clinical interpretation and highlights the need for future studies using standardized categories.

Regarding sialadenitis, the evolution of combined estimates over time appears less clear, with the estimated prevalence being relatively high in the months following treatment, decreasing over the course of the first year, and rising again thereafter (Fig. 3). However, our models were not adjusted for the administered activity, which varies widely between studies, with particularly high activities reported in studies with follow-up beyond 1 year (30, 39). Several studies have shown that the cumulative I^131^ activity is associated with an increased risk of sialadenitis, which could contribute to the slightly higher prevalence observed after 1 year compared with the 6–12-month period. Finally, the absolute difference in prevalence between the 6–12-month period (20%) and follow-up beyond 1 year (24%) remains small, with overlapping confidence intervals, making it difficult to conclude that there is a true re-increase in risk rather than fluctuations related to inter-study heterogeneity. The authors distinguish an acute phase of sialadenitis, occurring within hours to weeks after iodine administration, which generally resolves with simple measures (hydration, sialagogues, massage) (21, 52), from a chronic form, which may appear or persist after the acute episode and may last for several years after RIT (53). Nevertheless, assessment tools differ considerably from one study to another (questionnaires, clinical examination, scintigraphy), as do the thresholds used to define sialadenitis (including or excluding mild forms).

Beyond the administered activity, certain individual factors influence the occurrence of these complications. Advanced age appears to be an independent risk factor for post-RIT salivary gland injury (39). Several studies also report a higher prevalence of salivary gland impairment in women compared to men, whether in the form of sialadenitis or glandular dysfunction (9, 39, 54). Furthermore, the thyroid cell stimulation method has been identified as a factor influencing the risk of salivary complications (48). Indeed, a lower incidence of xerostomia and sialadenitis in patients stimulated with rhTSH compared with those prepared by hormone withdrawal has been reported (48). A recent systematic review also suggests that preparation with rhTSH may reduce radiation-related salivary damage compared with hormone withdrawal (55). However, our meta-analyses were not adjusted for these potential confounders due to an insufficient number of studies reporting this information, which likely contributed to the substantial inter-study variability. Future research should, therefore, include multivariable analyses that take into account age, sex, hormonal preparation method, baseline salivary function, and comorbidities in order to refine risk estimates. Finally, it should be noted that data on salivary and lacrimal complications in pediatric or young adult populations remain scarce, with only two studies in this review (17, 37). In fact, given the high survival rate for thyroid cancer (1), treatment side effects are particularly likely to have a lasting impact on quality of life. Future research should, therefore, aim to better characterize the long-term impact of I-131 therapy on salivary and lacrimal gland function in these younger populations.

Although less extensively studied, some findings regarding lacrimal gland involvement after RIT are available in the literature. Lacrimal gland disorders, such as xerophthalmia, are less common but still important, as noted in several studies (16, 18, 27, 34). Cases of xerophthalmia have been reported several months after the administration of I^131^. Yartsev *et al.* (56) demonstrated a correlation between I^131^ uptake by the lacrimal ducts and the reduction in tear secretion observed shortly after treatment. In addition, Jonklaas *et al.* (57) highlighted the potential – and sometimes significant – impact of these disorders on quality of life, despite their lower frequency compared with salivary gland involvement. However, the limited number of available studies does not allow for a meta-analysis. Additional standardized research is needed to better characterize the frequency, duration, and contributing factors of these adverse effects. The current lack of data underscores the importance of incorporating ocular follow-up in future studies to clarify the impact of treatment on the lacrimal glands, which remains poorly documented.

To our knowledge, this work is one of the first to provide both a qualitative and quantitative synthesis of the studies regarding the salivary and lachrymal troubles that may occur after RIT, according to different follow-up periods. This synthesis seems essential to reinforce the conclusions, which will be used to inform clinicians and patients, but also to assess the need for further studies. Our study highlighted the methodological heterogeneity of the available studies, and calls for further studies, particularly on the evolution of disorders before and after long-term treatment, using statistical models adjusted for risk factors in order to obtain more reliable estimates.

One strength of this work lies in the rigorous methodological approach adopted, in accordance with PRISMA guidelines, and in the inclusion of a wide range of studies published over more than 20 years. This approach provides an up-to-date overview of salivary and lacrimal adverse effects following RIT for DTC. Furthermore, combining a systematic review with complementary meta-analyses made it possible to quantify observations from qualitative analyses, thereby enhancing the robustness and consistency of our conclusions. No study was excluded from the meta-analyses for methodological reasons. However, the quality of the included studies was assessed using the Newcastle–Ottawa Scale (NOS), with most studies demonstrating good quality (score ≥ 6/9). However, depending on the meta-analysis, Egger’s tests were sometimes statistically significant, highlighting a publication bias or an asymmetry related to the high heterogeneity of long-term studies (Figs S2 and S3).

This review presents some limitations. First, most of the included studies are observational and retrospective, with a non-negligible risk of selection and confounding bias. The absence of comprehensive prospective longitudinal follow-up prevents a precise determination of the duration and progression of post-treatment disorders. In most longitudinal studies, it is not clearly established whether patients presenting late symptoms are the same individuals who were initially affected, making it difficult to distinguish between persistent and new cases. Second, the variability in assessment tools, follow-up durations – extended from a few days to more than 10 years –, and cumulative administered activity – from 0.74 GBq up to 93 GBq – complicates direct comparisons, maybe explaining the strong inter-study heterogeneity observed and limiting the precision of combined estimates, despite using random-effects models to account for heterogeneity. Third, objective methods (scintigraphy and sialometry) do not consistently align with subjective measures (questionnaires or symptom scales), making comparison of results more challenging. Standardization of the tools used to assess salivary and lacrimal gland function would strengthen the comparability of future studies. Pooled analyses based on individual-level data, rather than grouping by classes of administered activity, would allow for a more precise analysis of the relationship between administered activity and adverse effects, and for the identification of an exposure threshold beyond which the risk of glandular toxicity increases significantly. Ideally, a meta-regression using continuous cumulative activity (e.g. per GBq increment) or stratified analyses based on standardized and clinically relevant dose categories would have allowed a more informative and comparable assessment of the dose–response relationship. Future studies could adopt harmonized dose classifications to enable a more precise evaluation of potential dose–toxicity relationships.

## Conclusion

These systematic reviews and meta-analyses confirmed that salivary and lacrimal dysfunctions are common adverse events of RIT in patients treated for DTC. These complications are often associated with the administered activity level of RIT and may sometimes become chronic, although their frequency appears to generally decrease over time. However, the meta-analyses showed methodological heterogeneity among studies. The standardization of cumulative activity classifications and the integration of potential confounding factors should be considered to better characterize glandular toxicity thresholds.

## Supplementary materials





## Declaration of interest

The authors declare that there is no conflict of interest that could be perceived as prejudicing the impartiality of the work reported.

## Funding

This work did not receive any specific grant from any funding agency in the public, commercial, or not-for-profit sector.

## Author contribution statement

This study was designed by SB, M-OB, and CB; SB and CB conducted the systematic search and selected the studies in accordance with PRISMA guidelines. Disagreements were reviewed and resolved by M-OB; SB performed the qualitative and quantitative analyses. The first version of the report was written by SB, M-OB, CBU, CL, and CB, who take responsibility for the reliability and accuracy of the data. All authors reviewed and approved the final version of the manuscript.
