# Supplementary material for: Salivary and lacrimal disorders in patients treated with radioiodine for differentiated thyroid cancer
Source: Eur Thyroid J. 2026 May 18;15(3):ETJ250402. doi: 10.1530/ETJ-25-0402 (PMC13193070; doi:10.1530/ETJ-25-0402)
Supplement: Supplementary file 1 [file supplementary_figures.pdf]

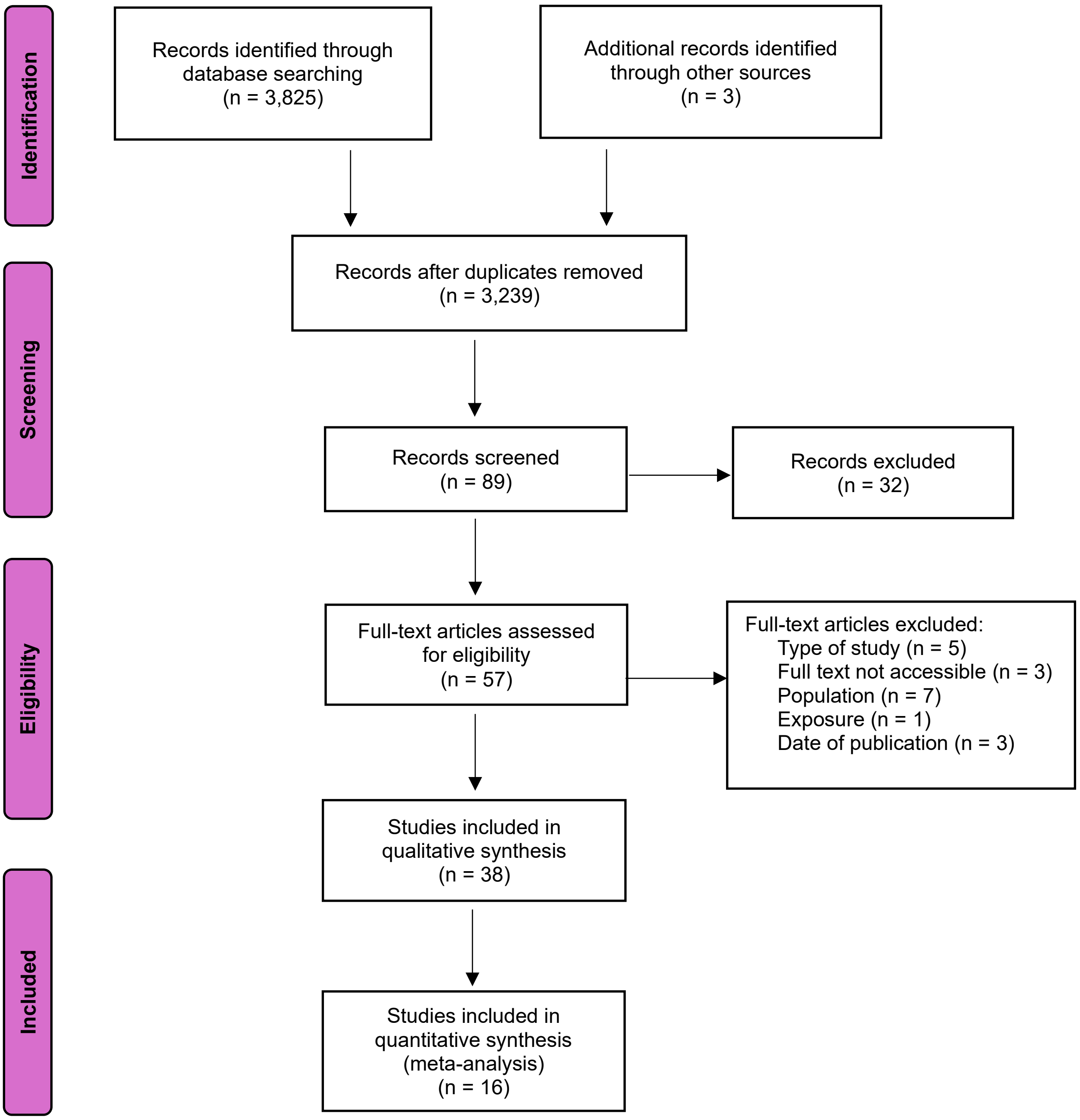

*Consider, if feasible to do so, reporting the number of records identified from each database or register searched (rather than the total number across all databases/registers).

**If automation tools were used, indicate how many records were excluded by a human and how many were excluded by automation tools.

Figure S1: The PRISMA flowchart

Group	Intercept	SE	z	p
< 2 months	-4.4634716	0.6405901	-0.7000720	0.6111690
2–6 months	-0.7124417	1.3904165	-0.1087936	0.9232979
> 6 months & < 1 year	1.2069048	0.7000934	0.3381673	0.7575196
> 1 year	-9.7323074	0.6383628	-3.1940358	0.0330885

Figure S2: Egger's test for meta proportions of xerostomia at different time post-RIT

```
Regression Test for Funnel Plot Asymmetry

Model:     mixed-effects meta-regression model
Predictor: standard error

Test for Funnel Plot Asymmetry: z = 0.8510, p = 0.3948
Limit Estimate (as sei -> 0):   b = 0.3369 (CI: -2.6668, 3.3407)
```

Figure S3: Egger's test for meta OR of xerostomia comparing low vs high activity administration

Group	Intercept	SE	z	p
< 2 months	-7.566312	0.009158705	-74.817698	0.00850844
> 6 months & < 1 year	-4.319264	0.451246830	-1.731503	0.33342046
> 1 year	-1.807450	0.303395798	-1.193685	0.44393697

Figure S4: Egger's test for meta proportions of sialadenitis
