# Supplementary material for: Salivary and lacrimal disorders in patients treated with radioiodine for differentiated thyroid cancer
Source: Eur Thyroid J. 2026 May 18;15(3):ETJ250402. doi: 10.1530/ETJ-25-0402 (PMC13193070; doi:10.1530/ETJ-25-0402)
Supplement: Supplementary file 2 [file supplementary_tables.pdf]

First author, year	Design	Outcomes	Measure tools	Time of assessment after RIT	Results	N<br>OS
**Albano et al. 2017[1]**	Retrospective cohort	Sialadenitis, xerostomia, gland dysfunction	Clinical exam, radiology, lab tests	≤ 1-week:<br><br>≥ few weeks:	**Xerostomia:** 14%<br>**Sialadenitis:** 22%<br>All late **adverse events:** associated with number of treatments and cumulative I^{131} ($p < .001$)	6
**Almeida et al. 2009[2]**	Cross-sectional	Pain, swallowing & saliva scores	Questions fromUW-QOL	Range: 4 months-10 years (median: 2 years)	Association with RIT <5.55 GBq: **pain** ($p = .045$); **swallowing** ($p = .03$); **saliva** score ($p = .20$)	7
**Almeida et al. 2011[3]**	Cross-sectional	Saliva flow, dysphagia	SGS (99mTcO$_4$), sialometry, questionnaire	Range: 2m to 10y (median: 24m)	**SSF** & **USF:** No significant effect of RIT ($p = .17$ and $p = .10$)<br>**Xerostomia:** No significant effect of RIT ($p = .63$)<br>**Dysphagia:** Significant effect of RIT ($p = .002$)<br>**Decreased excretion**: significant effect of RIT in **PG** ($p < .001$) and in all salivary glands combined ($p = .002$)<br>**Uptake** or **elimination** ability: No significant effect of activity (≤5.55 vs. >5.55 GBq)<br>Persistent **taste disturbance**: 15.6% of which 67.8% RIT	8
**Badam et al. 2016[4]**	Prospective cohort	Salivary flow, uptake, ejection fraction	SGS (99mTc-pertechnetate)	Baseline vs. 1 month	**%uptake:** (Group A & B) reduced in PG/SMG (p≤.001)<br>**Ejection fraction:** (Group A) No significant lower value in PG/SMG on right and left sides; (Group B) reduced in PG/SMG (p≤.001)	7
**Baudin et al. 2023[5]**	Prospective cohort	Xerostomia, dry eyes, hyposalivation, parotid pain, saliva flow, saliva biochemistry	Saliva sampling, biochemistry, OSDI, HAD, SF-36	T0: Baseline<br>T6: 6 m	**Xerostomia:** increased from 17.7% to 31.6% (22% new at T6); OR = 1.43 (95%CI 1.02–2.04) for 1-Gy; associated with I^{131} ($p = .04$)<br>**Stimulated flow:** decreased significantly at T6; −0.08 mL/min (CI −0.12 to −0.02) for 1-Gy<br>**Unstimulated flow:** not significantly changed<br>**Potassium concentration**: +1.07 mmol/L (CI 0.42–1.71) for 1-Gy<br>**Xerophthalmia:** increased from 19.1% to 28.2% (17% new at T6); associated at 1.1 GBq (OR = 3.58, 95%CI1.19–10.81), not at 3.7 GBq	8
**Bulut et al. 2018[6]**	Prospective cohort	Xerostomia	PROM, XI, XQ	Baseline vs. 3 months after sialendoscopy or postoperatively	**XQ** & **XI** scores: Improved (interventional group) $p = .006$ & $p = .005$; No significant changes (control group)	7
**Caglar et al. 2002[7]**	Cross-sectional	Salivary dysfunction, xerostomia	SGS (Tc-99m), visual scoring	Mean: 20 m (range: 5-250 m)	**Xerostomia:** 54%; association with I^{131} ($p = .006$)<br>Objective **dysfunction**: 69%; PG more affected than SMG (81% vs. 13%, $p < .001$); association with I^{131} ($p = .007$)	7
**Chow et al. 2021[8]**	Retrospective matched-pair	Hypocalcemia, dysphonia, dysphagia, xerostomia	Online self-survey, PROMIS-29	Mean ≈3y:<br><br>Months to years:	**Dysphonia:** 38% (RIT) vs. 44% (no RIT), $p = .44$<br>**Dysphagia:** 34% (RIT) vs. 27% (no RIT), $p = .27$<br>**Xerostomia:** 17.2% moderate to severe (RIT)	6
**Daniel et al. 2018[9]**	Prospective cohort	Xerostomia, dysphagia	Stimulated saliva, questionnaire, spectrophotometer	M1: 30-45d before<br>M2: 1-2d<br>M3: 7-10d	**SF:** 22 patients reduced at M2 and 7 patients recovered normal at M3 (no statistically significant)<br>**Hyposalivation** (<0.7 mL/min): 35% at M1, 40% at M2, 27% at M3<br>**Calcium** & **phosphate:** Significant reduction M1 vs. M2 ($p = .023$ & $p = .018$)<br>**Xerostomia:** 42% (M1), 68% (M2), 73% (M3), M1 vs. M2 : $p = .013$, M1 vs. M3 : $p = .251$	5
**Dingle et al. 2013[10]**	Cross-sectional survey	Association of RIT dose with prevalence of sialadenitis	MDADI, UW-QOL, XeQOLS		**Swallowing issues**: 31.7%; No correlation swallowing–activity ($R^2 = 0.092$, $p = .276$)<br>**Salivary gland issues**: 11.9% (<5.55 GBq); 23.8% (>5.55 GBq); $R^2 = 0.217$ & $p = .00965$ for activity-salivary problems<br>**Sialadenitis:** 20% overall; No sialadenitis ≤3.7 GBq; 26% (3.7-7.4 GBq); 43% (>7.4 GBq); OR = 2.47 ($p = .04$) for >5.55 GBq; $R^2 = 0.335$ & $p < .001$ for activity–sialadenitis	6
**Eiras Ramim et al. 2019[11]**	Prospective cohort	Xerostomia, pain, swallowing & saliva score	Questions from EORTC QLQ-C30, EORTC QLQ-H&N35	Baseline vs. 1-week:<br><br><br>Baseline vs. 3-months:	**Mouth/throat pain** worsening: $p < .001$<br>**Xerostomia** worsening: $p = .007$<br>**Sticky saliva** worsening: $p = .001$<br>Persistent **mouth/throat pain**: $p = .024$<br>Difficulty **swallowing**: $p = .002$	8
**Florenzano et al. 2016[12]**	Prospective cohort	Xerostomia (early/late)	Online survey, Likert scale	Within 6m (median; 2.5m, IQR; 0.5–5.8):<br><br><br><br><br>At least 6m (median; 11.5m, IQR; 6–18.6):	**RIT vs. no RIT:**<br>Salivary gland **pain:** 58.1% vs. 12.5% ($p < .01$)<br>**Swelling:** 55.8% vs. 16.7% ($p = .001$)<br>**Xerostomia:** 60.5% vs. 29.2% ($p = .007$)<br>**Dysgeusia:** 65.1% vs. 12.5% ($p < .01$)<br>**Smell** impairment: 30.2% vs. 4.2% ($p = .009$)<br>Salivary gland **pain:** 63.3% vs. 8.3% ($p = .001$)<br>**Swelling**: 49% vs. 16.7% ($p = .042$)<br>**Xerostomia:** 71.4% vs. 33.3% ($p = .014$)	6
**Geres et al. 2015[13]**	Retrospective cohort	Salivary response, pain, swelling	SGS (99mTc), clinical exam	Mean: 5 ± 3 m	**Sialadenitis:** 35%; pats. without sialadenitis more likely received single RIT (61%; $p = .001$)	5
**Goswami et al. 2019[14]**	Cross-sectional	Xerostomia, sialadenitis, xerophthalmia	Question from PROMIS 29-item	Days and weeks:<br><br><br><br>Months to years:	**Xerostomia:** mild 34%, moderate 25.3%, severe 14.1%<br>**Sialadenitis:** mild 26.5%, moderate 19.8%, severe 11.8%<br>**Taste/appetite changes:** 29%<br>**Xerophthalmia:** mild 23.5%, moderate 15.2%, severe 6.4%<br>**Xerostomia:** mild 24.2%, moderate 15.4%, severe 10%<br>**Xerophthalmia:** mild 23.8%, moderate 12.4%, severe 7.3%	7

**Grewal et al. 2009**[15]	Retrospective cohort	Xerostomia, taste change, gland pain/swelling	Medical records, clinic visits, phone calls	Between 2w and 1y:<br><br>Median of 7y:	**Salivary side effects:** 39%; Activity >2,78 GBq increased risk<br>**Xerostomia:** 17%, **Swelling:** 16%, **Pain:** 6%, **Altered taste:** 13%<br>**Xerostomia:** 13% did not recover	6
**Hedman et al. 2017**[16]	Cross-sectional	Xerostomia, dysphagia	Specific questionnaire	14–17 years after diagnosis	**Xerostomia:** 16%<br>**Dysphagia:** 10%<br>**Gland problems:** 4%	7
**Hollingsworth et al. 2016**[17]	Retrospective cohort	Xerostomia, sialadenitis	Self-report, medical coding validation	Mean: 6.29 y (median: 4.16 y; range: 2 w to 35 y)	**Xerostomia:** scores higher in RIT vs. no RIT (P = .002); OR = 4.01 (95%CI, 1.81–8.90; P = .001) RIT vs. no RIT, HR = 2.01 (95%CI, 0.98–4.13; P = .058); higher cumulative ^{131}I (mean difference: 1.7 GBq, 95% CI, 15–77; P = .003); first-administered activity not correlated; patients without prior sialadenitis: 25.9% (RIT) & 8.5% (no RIT)<br>**Sialadenitis:** HR = 7.43 (95%CI, 1.67–33.01; P = .008) RIT vs. no RIT; 57.7% with prior sialadenitis vs. 25.9% without prior disease, OR = 4.10 (95%CI, 1.59–10.54; P = .003); no difference in cumulative or first-administered ^{131}I activity between patients with or without recurrence	7
**Horvath et al. 2020**[18]	Retrospective cohort	Salivary damage, taste alteration, xerostomia	Ultrasound, self-questionnaire	≥ 12 m:<br>12–60 m:	**Salivary gland damage**: 25.1%; OR = 5.19 (standard error: 0.92, p < .001) for cumulative I^{131}<br>**Taste** alteration 35%, **painful** parotid 37%, **dysphagia** 16%, **lacrimal** issues 15%	8
**Huang et al. 2021**[19]	Retrospective cohort	Salivary changes, xerostomia	Ultrasound, dry mouth criteria, RTOG/EORTC	Median: 50 m (range: 32–73)<br>Salivary gland ultrasound every 3m to 6m after the last RIT	**Xerostomia:** 23% mild;10% moderate to severe<br>Effect of cumulative I^{131} on f/u ultrasound positivity:<br>≤5.55 GBq: significantly lower rate (OR = 0.063, P < 0.001)<br>≤11.47 GBq: significantly lower rate (OR = 0.279, P < 0.001)<br>≤12.95 GBq: no significant difference (OR = 0.641, P = 0.133)<br>12.95 GBq: highest risk (reference group)	7
**Hyer et al. 2007**[20]	Prospective cohort	Salivary toxicity (pain, swelling, dryness)	Patient reports, hospital records	F/u 6w, then every 8–12w, and at least annually for 4 years	Salivary gland toxicity (**xerostomia**, **swelling** & **pain**): 15% within 48h; 12% at 3m; 21% at 12m<br>**Gland involvement:** 50% SMG; 39% PG; 11% both glands	7
**Iakovou et al. 2015**[21]	Nonrandomized, controlled, open-label prospective	Xerostomia, sialadenitis	XI, massage incidence, whole-body scan	1m & 12m	**Salivary gland dysfunction** (**sialadenitis** & **xerostomia**)**:** 33% (1m); 12% (12m); Lower dysfunction in rhTSH vs. LT4 withdrawal group (p = .002 & .021); No significant correlation with I^{131} activity<br>**Xerostomia:** 16% (rhTSH) vs. 46% (LT4 withdrawal) at 1m; 0.016% (rhTSH) vs. 22% (LT4 withdrawal) at 12m	8
**Jeong et al. 2013**[22]	Prospective cohort	Salivary dysfunction/uptake/ejection fraction, xerostomia	SGS (99mTc), patient interviews	1-2w before vs. 5y	**Xerostomia:** 16% at 5y f/u<br>**PG** more frequently affected than **SMG**<br>**Worsening excretion fraction**: 44% (5.55 GBq) vs. 16% (3.7 GBq), p = .03<br>**Worsening U score:** 49% (5.55 GBq) vs. 18% (3.7 GBq), p = .02; higher average activity (5.49 ± 6.59 GBq vs. 5.07 ± 8.18 GBq, p = .04)<br>Activity >5.55 GBq: more severe **dysfunction** than 3.7 GBq	6
**Klein Hesselink et al. 2016**[23]	Prospective cohort	Saliva flow, xerostomia	Sialometry, sialochemistry, XI, whole-body imaging, SPECT/CT scans	1-week before vs. 5.3 ± 0.6 m	**Unstimulated flow:** no changes for pats. with repeated RIT<br>**Stimulated flow:** PG + SMG decreased; higher cumulative I^{131} correlated with ≥50% reduction (p = .026); In patients with repeated I131 treatment (n = 11), no changes for pats. with repeated RIT<br>**Xerostomia:** 4% before & 20% after RIT; scores increased (p = .001); no change XI score (p = .064)	7
**Krčálová et al. 2020**[24]	Prospective cohort	Salivary uptake/excretion, hyposialia	SGS (99mTc), RTOG-modified UW Head & Neck Scale	Baseline vs. 4–6 m :	Salivary gland **uptake** and **excretion fractions** (PG or SMG): no significant differences	7
**Le Roux et al. 2020**[25]	prospective comparative within-population	Xerostomia, pain, swelling	Questionnaire, VAS	Over the last 3 years	**Xerostomia:** 33% persistent<br>**Discomfort** & **swelling:** 27% (2013) to 14% (2019)<br>**Pain PG:** 23% to 11%<br>**Bad taste:** 10% to 4%<br>31% recovered from **salivary side effects** late in f/u	7
**Lee et al. 2015**[26]	Retrospective cohort	Salivary dysfunction, imaging correlation	Interview, ultrasound, CT	Immediately, <6m, 6–12m, >12m	**Salivary gland dysfunction:** 46%; higher RIT increased risk (p < .001)<br>**Xerostomia:** 54%<br>**Swelling** & **pain**: 61% & 24%<br>Onset of symptoms: Immediately (29%), within 6m (37%), 6–12m (21%), after 12m (13%)	6
**Lopes da Fonseca et al. 2017**[27]	Prospective cohort	Xerostomia	OSDI, NOS, TBUT, XI, Schirmer, Rose Bengal, nasal endoscopy & saliva test	Before surgery vs. 1m post-surgery and 2, 4, 6, and 12m post RIT	**Xerostomia** & **xeropthalmia:** Higher subjective inventory & OSDI scores in RIT grp.<br>**SSF** & **USF:** Significant decrease vs. preoperative & postoperative values (p < .01) in RIT grp.; Significant decrease (only at 4m) vs. preoperative values (p = .02) in control grp.<br>**SSF RIT vs. No RIT: significant decreased** at 2m (p = .02), 4m (p = .02), and 6m (p = .01)<br>**USF RIT vs. No RIT:** significant differences in saliva production before stimulation at 4m (p < .01)	8
**Makarenko et al. 2024**[28]	Retrospective cohort	Pain, swelling, xerostomia, taste change	I131 scan, ultrasound, patient reports	Few days to 6 months & more than 6 months	**Xerostomia:** 38% (8.2% persistent, 10.7% permanent)<br>**Sialadenitis:** 51.2% (44.2% resolved, 27.6% chronic)<br>**Discomfort or pain:** 51.2% (28.5% in first days/weeks, resolved)<br>**Salivary gland swelling:** 50.2% (27.8% single early episode & resolved, 16.4% recurrent, 6% recurrent & prolonged)	6
**Maruoka et al. 2017**[29]	Retrospective cohort	Salivary gland dysfunction	SGS (99mTc-pertechnetate), functional scoring	12±1 months	**Functional scores:** lower in ≥10 GBq vs. pre-treatment & <10 GBq (p < .001)<br>**Xerostomia:** 15.4% (43/279); 10 (<10 GBq), 33 (≥10 GBq); Associated with cumulative 131I activity (p < .001); PG/SMG functional scores independent risk factors (OR = 0.03 & 0.0007)	8

**Ming et al. 2022[30]**	Prospective cohort	Xerostomia	Question from EORTC QLQ-C30 & EORTC QLQ-THY34	Day 3 vs. day −1:<br>1 month vs. day -1:	**Xerostomia** worsening: p = .018<br>**Xerostomia** improvement: p = .585	6
**Missaoui et al. 2022[31]**	Cross-sectional	Sialadenitis	Clinical observation	Mean: ≈5y	**Sialadenitis:** 22.1%<br>RIT complications: 26.7%	7
**Nabaa et al. 2012[32]**	Retrospective cohort	Salivary dysfunction (CT correlation)	SGS (99mTc-pertechnetate), non-contrast CT	Baseline (range: 1–15d)<br>Mean: 9m (range: 6–12m):	**PG**: 19.5% volume reduction (sensitivity: 86%, specificity: 100%); attenuation increased with dysfunction grade (p < .001)<br>**SMG**: 31.0% volume reduction (sensitivity: 100%, specificity: 97%)	6
**Rogers et al. 2017[33]**	Cross-sectional	Xerostomia	Question from EORTC QLQ-C30	Median: 31 (IQR: 19–46) months	**Xerostomia:** 31% 'quite a bit' or 'very much'	5
**Selvakumar et al. 2019[34]**	Cross-sectional	Salivary dysfunction, xerostomia	Sialometry, sialochemistry, XI	Median: 11 y (IQR: 6–22 y)	**SSF:** activity ≥7.4 GBq associated with lower (r = .287, p = .023); Potassium & amylase levels associated with I^{131} activity & number of RIT<br>**USF:** No significant associations with I^{131} activity (r = .057, p = .655)<br>**SGD:** Multiple RIT: (OR = 3.00, 95%CI 1.07–8.39; p = .036); Associated with cumulative I^{131} activity (p = .05) & (OR = 1.32, 95%CI 1.09–1.61)<br>**Xerostomia:** 36% moderate to severe; Higher XI scores in multiple RIT (p = .025) and cumulative activity ≥3.7 GBq (p = .041)	8
**Solans et al. 2001[35]**	Prospective cohort	Salivary/lacrimal dysfunction, xerostomia	SGS (99mTc), Schirmer, Rose Bengal, TBUT	First-year f/u:<br><br>Second-year f/u:<br>Third-year f/u:	**Xerostomia:** 33% (p < .001 vs. baseline); **Salivary dysfunction:** 51% (p < .001 vs. baseline); **Xerophthalmia:** 25% (p < .001 vs. baseline); **Lacrimal dysfunction:** 18%<br>**Xerostomia:** 20% (p = .002 vs. year 1); **Xerophthalmia:** 18% (p = .03 vs. year 1).<br>**Xerostomia:** 15%; **Xerophthalmia:** 14%<br>Activity >11.1 GBq correlated with severe dysfunction; Higher cumulative activity correlate with salivary gland dysfunction (p = .049)	7
**Tabari et al. 2024[36]**	Prospective cohort	Smell, taste, saliva changes	VAS, Self-MOQ, UW-QOL, smell test	Baseline, 1 m, 6 m	Decline in **smell**: p = .015 (1 month), p = .031 (6 months)<br>Decline in **saliva** (p = .001)<br>**Taste:** improved from 1-m to 6-m f/u (p < .001)	6
**Walter et al. 2007[37]**	Longitudinal cohort	Sialadenitis, xerostomia	SGS (gamma camera), dental follow-up, CTCAE	Median: 6,6 y (range: 1,1–32,6 y)	**Xerostomia:** 44.3%; OR = 1.15, 95%CI 1.04–1.29, (p = .01) per GBq; OR = 2.88, 95%IC1.64–5.07, (p = .001) if sialadenitis<br>**Sialadenitis:** 24.4%; OR = 1.03, 95%IC 0.94–1.13, (p = .53) per GBq	7
**Wu, Feng et al. 2015[38]**	Retrospective cohort	Salivary uptake/secretion reduction, xerostomia	SGS (99mTcO), gland function score	Range: 1–22 m	**Xerostomia:** 43.7%; severity correlated with number of cycles and I^{131} activity (r = 0.220, 0.246; p = .014, 0.006, respectively)	9

Abbreviations: SSF stimulated salivary flow; USF unstimulated salivary flow; Pts. Patients; F/u follow-up; d day, w week, m month, y year; DTC differentiated thyroid carcinoma; RIT radioiodine therapy; GBq gigabecquerel; T thyroidectomy; SGS salivary gland scintigraphy; rhTSH recombinant human Thyroid-Stimulating Hormone; SGD salivary gland dysfunction; SMG submandibular gland; PG parotid gland

Table S1: Characteristics and results of the included studies and outcomes

**First author, year, country**	**Population / Inclusion criteria**	**RIT Number of patients treated, activity (GBq), number of RITs**	**Conclusion**	**Advantages / limits**
**Albano et al. 2017[1]<br>Italy**	28M, 77F; 92 papillary, 13 follicular / ≤18y; T+ RIT	50 pts.: 1 RIT, 55 pts.: ≥2 RITs<br>1.1-93.4 (mean: 16.2)	Activity related early/late complications; cumulative I^{131} effect; no significant associations with age and sex	Both early/late analysis; pediatric focus / small sample; retrospective; single center

**Badam et al. 2016[2] India**	24 DTC / T + RIT		Dose-dependent gland decline; parotid > submandibular	Quantitative scintigraphy; confirms dose–damage link / small sample; no long-term follow-up
**Baudin et al. 2023[5] France**	39M, 97F; 116 papillary, 20 follicular<br>97.8% participation / T; awaiting first RIT	44 pts.: 1.1<br>92 pts.: 3.7	Mild xerostomia/dry eyes; no major dysfunction	First dosimetry-based; objective + subjective measures; adjusted analysis / few cases; subjective–objective mismatch
**Bulut et al. 2018[6] Germany**	2M, 10F; 10 papillary, 2 follicular / xerostomia after RIT	7 pts.: 1 RIT (3.7–5.5)<br>5 pts.: ≥ 2 RITs (6.3–18.4)	I^{131} activity not predictive; sialendoscopy improves outcomes	Significant patient improvement; safe / small sample; short f/u (3m); single-center; nonrandomized; no standard protocol
**Daniel et al. 2018[9] Brazil**	6M, 31F; DTC / RIT	3.7-9.25	Early xerostomia/dysphagia after RIT; partial recovery by 3m	Controlled before–after; individual tracking / small sample; short f/u; loss to f/u
**Eiras Ramim et al. 2019[11] Brazil**	30M, 102F; 108 papillary, 24 follicular.<br>4 pts. lost to f/u / T + RIT under TSH	29 pts.; 1.11-1.85 / 91 pts.; 3.70-5.55<br>12 pts.; 7.40-9.25	RIT effective, well tolerated; no major long-term effect	Validated instruments; no THW; low dropout (3%) / single center; nonspecific questionnaire; comorbidities not assessed
**Florenzano et al. 2016[12] Chile**	110 (91.8% F); DTC.<br>61 participated in both surveys / T	86 pts.: 1.11–5.55 / 38 pts.: ≤1.85<br>48 pts.: ≥ 3.7	RIT symptoms frequent/persistent (>6m), even low dose (1.85 GBq)	Prospective; early/late data; focused symptom survey / no validated tool; selection bias (~50% f/u); hypothyroidism confounding
**Geres et al. 2015[13] Argentina**	7M, 45F; DTC / T + RIT	1.1-5.5 per RIT	No effect of cumulative I^{131} for sialadenitis incidence	Identified risk factors; effective intraductal therapy /small sample; no long-term data
**Grewal et al. 2009[15] United States**	262 (66% F); 93% papillary / T + RIT	1.07-16.65 (mean: 5.25, median: 5.21)	Most salivary effects resolve; <1% persistent pain/swelling and 2% xerostomia; high increased risk	Large long-term cohort (7 y); dose–response shown / retrospective; recall bias; no objective data
**Hollingsworth et al. 2016[17] United States**	45 & 386M, 171 & 1121F (initial/ validation); 194 & 1315 papillary, 19 & 183 follicular, 3 & 9 others	0.93-31.45 initial study<br>0.74-44.0 validation study	I^{131} activity, gender, history of sialadenitis before treatment were risk factors for salivary gland damage	Large + validation cohort; multi-source data / retrospective; recall bias; missing records; no objective test
**Horvath et al. 2020[18] Chile and Nicaragua**	135M, 435F; papillary / RIT	77 pts. (grp.A): 1.11-1.3 / 79 pts. (grp.B): 1.78-2.15 / 219 pts. (grp. C): 3.66-4.07 / 177 pts. (grp. D): 5.55-5.92 / 18 pts. (grp. E): 7.4-18.5	Chronic sialadenitis risk directly proportional to the cumulative I^{131} activity	Largest USG-based study; clinical impact / single center; incomplete QoL data
**Huang et al. 2021[19] China**	123M, 323F; DTC<br>89.6%, 58% and 19.5% participation for 5th, 6th and 7th f/u respectively **/** T + RIT	52 pts. (grp. 1): ≤5.55 / 161 pts. (grp. 2): 5.58–11.43 / 132 pts. (grp. 3): 11.47–12.95 / 101 pts. (grp. 4): 12.95–23.23	Progressive irreversible gland changes; ≤11.47 GBq safer	Complete long-term US follow-up; age effect / low follow-up rates; no pathology; no sublingual eval
**Hyer et al. 2007[20] United Kingdom**	19M, 57F; 56 papillary, 18 follicular, 2 Hürthle / T + RIT	8.5-17.7 (median: 14)<br>3 (first RIT) / 5.5 (if second RIT)	Salivary toxicity common/persistent after RIT; long-term	Long f/u (4 y); large database / no objective tests; dose variability; prevention need
**Jeong et al. 2013[22] South Korea**	213 (90.6% F); 95.8% papillary, 4.2% follicular<br>Thyroidectomy + RIT; f/u ≥ 52m after RIT	3.7–5.55 (mean: 5.1)	~20% glands dysfunctional at 5 y; higher activity linked to more severe dysfunction	Large sample; pre & post SGS comparison; long-term (5 y); objective + subjective / high doses; hypothyroid confounding
**Klein Hesselink et al. 2016[23] The Netherlands**	25M, 42F; 54 papillary, 7 follicular, 6 Hürthle<br>70.5% participation	3.7 or 5.5<br>56 pts.: 1 RIT / 11 pts.: several RITs	High activity I^{131} decreased salivary function	Prospective, pre/post; multiple measures / hormone status confounder; small repeated subgroup
**Krčálová et al. 2020[24] Czech Republic**	6M, 25F; 28 papillary, 2 follicular, 1 Hürthle<br>T + RIT	3.7	RIT with 3.7 GBq does not appear to cause significant salivary gland dysfunction	Objective scintigraphy; prospective; adequate power / small sample; short f/u (6m)
**Le Roux et al. 2020[25] France**	36M, 126F; DTC<br>79% participation	3.7	Persistent xerostomia; gradual recovery after 6y	First long-term trend analysis; high response (78%) / recall + nonresponse bias; no objective measures
**Lopes da Fonseca et al. 2017[27] Brazil**	12M, 75F, (44 and 43 in grp. 1 and 2); 71 papillary, 16 follicular.<br>86.4%, 81.4% participate (grp. 1, 2)	3.74-9.87 (mean: 7.09)	Saliva production decreased (activity related); ocular unchanged	Objective + subjective measures; antioxidant/NIS-blocker suggestion / small sample; seasonal confounding
**Makarenko et al. 2024[28] Ukraine**	57M, 273F; DTC<br>More than 1y f/u after the initial RIT	64 pts.: <3.7 / 207 pts.: 3.7–5.55<br>59 pts.: >5.55	Chronic radiation sialadenitis common; activity main risk	Long-term insight; prevention need / retrospective; variable f/u; recall bias
**Maruoka et al. 2017[29] Japan**	78M, 201F; 258 papillary, 18 follicular, 3 papillary and follicular. 77% participation	3.7-33.9<br>193 pts.: <10 / 86 pts.: ≥10	PG/SMG function were main predictors of xerostomia; activity not	SGS detects dysfunction pre-symptom; predictive functional scores / no saliva volume data; retrospective; variable uptake
**Ming et al. 2022[30] China**	27M, 72F; 97 papillary, 2 follicular<br>T + RIT under THW	3.7 or 5.55	RIT cause salivary gland damage	First use of EORTC QLQ-C30 & THY34 in RIT / 1m f/u only; single-center (regional bias)
**Nabaa et al. 2012[32] Japan**	13M, 27F; DTC<br>T + RIT	5.14–5.55 (mean: 5.35)<br>20 pts.: 1 RIT / 20 pts. ≥2 RITs	CT predicts/grades RIT dysfunction; parotid > submandibular	Activity dependent dysfunction; high sensitivity & specificity; small sample; timing uncertain
**Solans et al. 2001[35] Spain**	11M, 68F; 65 papillary, 11 follicular, 2 Basedow's disease	0.93–18.5 (first RIT)<br>2.96–5.55 (if second RIT)	Frequent salivary/lacrimal dysfunction after RIT; mostly resolves	Long prospective f/u; objective + subjective data; first combined study / small sample; no control; dose variability
**Tabari et al. 2024[36] Iran**	17M, 81F; 94 papillary, 4 follicular	Mean: 5.38 ± 1.15	Temporary smell/taste/saliva changes; recovery by 6m	Combined subjective + objective tools; diverse measures / loss to f/u; limited saliva/taste testing
**Walter et al. 2007[37] Switzerland**	50M, 126F; 110 papillary, 65 follicular, 1 unknown. 87.1% participation / T	1.9–35.0 (median: 7.4)<br>1–6 RITs (median: 2)	High cumulative I^{131} activity affect long-term dental health	Long-term registry; dentist assessments / no scintigraphy; selection bias (refusals)
**Wu, Feng et al. 2015[38]**	95M, 273F; 362 papillary, 6 follicular / T + RIT	174 pts. (grp. 1): 0/ 78 pts. (grp. 2): 1.85–5.55/	Activity ≤5.55 GBq not significantly affect	Large sample; quantitative scoring / short f/u;

**China**		35 pts. (grp. 3): 5.58–11.1/ 48 pts. (grp. 4): 11.14–22.2/ 33 pts. (grp. 5): >22.2	gland function; >22.2 GBq cause severe dysfunction, especially in the PG; Pain/swelling rare and temporary	unbalanced dose groups

Abbreviations: M & F Male & Female; Pts. Patients; F/u follow-up; d day, w week, m month, y year; DTC differentiated thyroid carcinoma; RIT radioiodine therapy; GBq gigabecquerel; T thyroidectomy; SGS salivary gland scintigraphy; rhTSH recombinant human Thyroid-Stimulating Hormone; SGD salivary gland dysfunction; SMG submandibular gland; PG parotid gland

Table S2: Characteristics of cohort studies included in the systematic review

First author, year, country	Population, Inclusion criteria	RIT Number of patients treated, activity (GBq), number of RITs	Conclusion	Advantages / limits
**Almeida et al. 2009[2] Brazil**	17M, 137F; 151 papillary, 3 follicular. 38.5% participation T; normal thyroid hormone levels	93 pts. 1.11–25.9 (median: 4.81) / 73 pts.: ≤5.55 / 20 pts.: >5.55	I^{131} activity >5.55 GBq affects pain, swallowing, chewing, speech, taste	Validated QoL; multivariate analysis / variable post-treatment timing; no control/pre-baseline; subjective data
**Almeida et al. 2011[3] Brazil, Colombia**	23M, 159F; 177 papillary, 5 follicular 45.5% participation T + RIT	106 pts. Range: 1.11–16.65 (median: 5) 82 pts.: ≤5.55 / 26 pts.: >5.55	Impaired PG excretion + dysphagia; RIT effect confirmed	Clinical proof; salivary dysfunction shown / retrospective; recall bias; no validated questionnaire
**Caglar et al. 2002[7] Turkey**	16M, 55F; 53 papillary, 11 follicular, 4 mixed, 3 Hürthle T + SGS	45 pts. 18 pts.: 3.7 / 16 pts.: 5.55 / 11 pts.: ≥7.4	Frequent salivary gland dysfunctions; xerostomia is the most common symptom	Objective imaging correlation / small sample; no randomization; limited power
**Chow et al. 2021[8] United States**	17M, 227F (122 matched pairs); 233 papillary, 9 follicular, 2 Hürthle Stage I DTC; t; completed both parts of the survey	122 pts.	No significant impact of RIT on dysphagia and dysphonia	Patient-reported outcomes; large diverse US cohort / self-report/recall bias; missing RIT details (activity, timing); possible selection bias
**Dingle et al. 2013[10] United States**	24M,121F; 99 papillary, 34 follicular, 9 micropapillary, 2 oxyphilic, 1 Hürthle. 38% participation	116 pts. 0-30.93 (mean: 5.72) 84 pts.; ≤5.55 / 58 pts.; >5.55	I^{131} activity >5.55 GBq main risk for sialadenitis; salivary dysfunction frequent	First to assess RIT–sialadenitis–QoL link; novel patient-reported outcomes / small sample; recruitment bias; non-validated baseline tool; comorbidities; recall bias
**Goswami et al. 2019[14] United States**	202M, 1541F; 1313 papillary, 97 follicular, 74 medullary, 42 Hürthle, 12 NIFTP, 7 anaplastic. 61% participation	1366 pts.	Common adverse effects caused by RIT	Large sample; broad data collection / selection & self-report bias; low diversity
**Hedman et al. 2017[16] Sweden**	61M, 218F; DTC 79% participation 18–50y at diagnosis, 35–64y at inclusion, T	105 pts.	Long-term thyroid-related symptoms persist	Nationwide, population-based; high response (79%) / self-report data; no general-pop control; non-validated questionnaire
**Iakovou et al. 2015[21] Greece**	20M, 101F; 111 papillary, 8 follicular, 2 Hürthle Tumor stages T1 to T3; no distant metastases or residual disease; T	30 pts. (Grp. A): rhTSH+3.7 / 31 pts. (Grp. B): rhTSH+2.6 / 29 pts.(Grp.C):LT4 withdrawal+3.7 / 31 pts.(Grp.D):LT4withdrawal+2.6	rhTSH prep reduces incidence of xerostomia/sialadenitis vs LT4; no activity link	Long-term effect evidence; supports rhTSH superiority / nonrandomized; no dynamic scintigraphy
**Lee et al. 2015[26] South Korea**	34M, 130F; 159 papillary, 5 follicular. 18.6% participation T + RIT; at least one f/u imaging > 6 months after RIT	46 pts.: 1.1 / 45 pts.: 3.7 / 62 pts.: 5.5 / 4 pts.: 7.4 7 pts.: >2 RITs	Gland dysfunction common after RIT; high activity increased risk	Long-term focus; dose correlation / retrospective; variable imaging/f/u; small sample; no early–late distinction
**Missaoui et al. 2022[31] Tunisia**	9M, 77F; 79 papillary, 7 follicular. Controls; 76 healthy individuals from the general population. 73.1% participation. Disease-free for at least 6m	Mean activity: 10.15	High cumulative I^{131} activityT worsens pain	Homogeneous sample; standardized treatment / no spiritual or thyroid-specific tool; no baseline HRQoL
**Rogers et al. 2017[33] United Kingdom**	69M, 180F pts. surveyed, 169 responded; 188 papillary, 54 follicular, 7 unknowns. 68% participation. RIT; disease-free	All pts.	HighI^{131} activity cause damage	First FoR screening / single institution; response bias
**Selvakumar et al. 2019[34] The Netherlands**	9M, 56F; 53 papillary, 12 follicular 62% participation ≤18y, at least 5y of f/u after RIT; ≥18y at evaluation	Median: 5.88 (IQR: 2.92–12.95) 34 pts.: 1 RIT (median: 3.7, IQR: 1.85–5.85) / 31 pts.: ≥2 RITs (median: 12.95, IQR: 7.92–18.50)	Long-term SGD in pediatric survivors; Higher cumulative ^{131}I activity associated with greater SGD and xerostomia	Quantitative, objective + subjective data; long f/u (11 y) / small sample; rare disease; retrospective; no pre-baseline; no control

Abbreviations: M & F Male & Female; Pts. Patients; F/u follow-up; d day, w week, m month, y year; DTC differentiated thyroid carcinoma; RIT radioiodine therapy; GBq gigabecquerel; T thyroidectomy; SGS salivary gland scintigraphy; rhTSH recombinant human Thyroid-Stimulating Hormone; SGD salivary gland dysfunction; SMG submandibular gland; PG parotid gland

Table S3: Characteristics of non-cohort studies included in the systematic review

1. Albano D, Bertagna F, Panarotto MB, et al. Early and late adverse effects of radioiodine for pediatric differentiated thyroid cancer. Pediatr Blood Cancer 2017;64(11); doi: 10.1002/pbc.26595.
2. Almeida JP, Vartanian JG, Kowalski LP. Clinical predictors of quality of life in patients with initial differentiated thyroid cancers. Arch Otolaryngol Head Neck Surg 2009;135(4):342–346; doi: 10.1001/archoto.2009.16.
3. Almeida JP, Sanabria AE, Lima ENP, et al. Late side effects of radioactive iodine on salivary gland function in patients with thyroid cancer. Head Neck 2011;33(5):686–690; doi: 10.1002/hed.21520.
4. Badam RK, Suram J, Babu DBG, et al. Assessment of Salivary Gland Function Using Salivary Scintigraphy in Pre and Post Radioactive Iodine Therapy in Diagnosed Thyroid Carcinoma Patients. J Clin Diagn Res 2016;10(1):ZC60-62; doi: 10.7860/JCDR/2016/16091.7121.
5. Baudin C, Bressand A, Buffet C, et al. Dysfunction of the Salivary and Lacrimal Glands After Radioiodine Therapy for Thyroid Cancer: Results of the START Study After 6-Months of Follow-Up. 2023.
6. Bulut OC, Haufe S, Hohenberger R, et al. Impact of sialendoscopy on improving health related quality of life in patients suffering from radioiodineinduced xerostomia. Nuklearmedizin 2018;57(4):160–167; doi: 10.3413/Nukmed-0964-18-03.
7. Caglar M, Tuncel M, Alpar R. Scintigraphic evaluation of salivary gland dysfunction in patients with thyroid cancer after radioiodine treatment. Clin Nucl Med 2002;27(11):767–771; doi: 10.1097/00003072-200211000-00003.
8. Chow KY, Kurumety S, Helenowski IB, et al. Association between quality of life and patient-reported complications from surgery and radioiodine in early-stage thyroid cancer survivors: A matched-pair analysis. Surgery 2021;170(2):462–468; doi: 10.1016/j.surg.2021.01.022.
9. Daniel FI, Lima LD, Grando LJ, et al. Salivary evaluation in radioactive I131 treated patients with thyroid carcinoma. Acta Odontologica Scandinavica 2018;76(2):148–152.
10. Dingle IF, Mishoe AE, Nguyen SA, et al. Salivary morbidity and quality of life following radioactive iodine for well-differentiated thyroid cancer. Otolaryngol Head Neck Surg 2013;148(5):746–752; doi: 10.1177/0194599813479777.
11. Ramim JE, Cardoso MAS, de Oliveira GLC, et al. Health-related quality of life of thyroid cancer patients undergoing radioiodine therapy: a cohort real-world study in a reference public cancer hospital in Brazil. Support Care Cancer 2020;28(8):3771–3779; doi: 10.1007/s00520-019-05225-x.
12. Florenzano P, Guarda FJ, Jaimovich R, et al. Radioactive Iodine Administration Is Associated with Persistent Related Symptoms in Patients with Differentiated Thyroid Cancer. 2016.
13. Geres AE, Mereshian PS, Fernández S, et al. Sialadenitis after radioiodine therapy. Analysis of factors that influence the response to medical treatment. Endocrinol Nutr 2015;62(10):493–498; doi: 10.1016/j.endonu.2015.07.006.

14. Goswami S, Peipert BJ, Mongelli MN, et al. Clinical factors associated with worse quality-of-life scores in United States thyroid cancer survivors. Surgery 2019;166(1):69–74; doi: 10.1016/j.surg.2019.01.034.
15. Grewal RK, Larson SM, Pentlow CE, et al. Salivary gland side effects commonly develop several weeks after initial radioactive iodine ablation. 2009.
16. Hedman C, Djärv T, Strang P, et al. Effect of Thyroid-Related Symptoms on Long-Term Quality of Life in Patients with Differentiated Thyroid Carcinoma: A Population-Based Study in Sweden. 2017.
17. Hollingsworth B, Senter L, Zhang X, et al. Risk factors of 131I-induced salivary gland damage in thyroid cancer patients. 2016.
18. Horvath E, Skoknic V, Majlis S, et al. Radioiodine-Induced Salivary Gland Damage Detected by Ultrasonography in Patients Treated for Papillary Thyroid Cancer: Radioactive Iodine Activity and Risk. Thyroid 2020;30(11):1646–1655; doi: 10.1089/thy.2019.0563.
19. Huang Z, Chen Y, Pan L, et al. Progressive changes in the major salivary gland after radioiodine therapy for differentiated thyroid cancer: a single-center retrospective ultrasound cohort study. Ultrasound Med Biol 2021;47(9):2514–2522; doi: 10.1016/j.ultrasmedbio.2021.05.013.
20. Hyer S, Kong A, Pratt B, et al. Salivary gland toxicity after radioiodine therapy for thyroid cancer. Clin Oncol (R Coll Radiol) 2007;19(1):83–86; doi: 10.1016/j.clon.2006.11.005.
21. Iakovou I, Goulis DG, Tsinaslanidou Z, et al. Effect of recombinant human thyroid-stimulating hormone or levothyroxine withdrawal on salivary gland dysfunction after radioactive iodine administration for thyroid remnant ablation. Head Neck 2016;38 Suppl 1:E227-230; doi: 10.1002/hed.23974.
22. Jeong SY, Kim HW, Lee S-W, et al. Salivary gland function 5 years after radioactive iodine ablation in patients with differentiated thyroid cancer: direct comparison of pre- and postablation scintigraphies and their relation to xerostomia symptoms. Thyroid 2013;23(5):609–616; doi: 10.1089/thy.2012.0106.
23. Klein Hesselink EN, Brouwers AH, de Jong JR, et al. Effects of Radioiodine Treatment on Salivary Gland Function in Patients with Differentiated Thyroid Carcinoma: A Prospective Study. J Nucl Med 2016;57(11):1685–1691; doi: 10.2967/jnumed.115.169888.
24. Krčálová E, Horáček J, Gabalec F, et al. Scintigraphic evaluation of salivary gland function in thyroid cancer patients after radioiodine remnant ablation. Eur J Oral Sci 2020;128(3):204–210; doi: 10.1111/eos.12689.
25. Le Roux M-K, Graillon N, Guyot L, et al. Salivary side effects after radioiodine treatment for differentiated papillary thyroid carcinoma: Long-term study. Head Neck 2020;42(11):3133–3140; doi: 10.1002/hed.26359.

26. Lee HN, An JY, Lee KM, et al. Salivary gland dysfunction after radioactive iodine (I-131) therapy in patients following total thyroidectomy: emphasis on radioactive iodine therapy dose. Clin Imaging 2015;39(3):396–400; doi: 10.1016/j.clinimag.2014.12.018.
27. da Fonseca FL, Yamanaka PK, Mazoti L, et al. Correlation among ocular surface disease, xerostomia, and nasal symptoms in patients with differentiated thyroid carcinoma subjected to radioiodine therapy: A prospective comparative study. Head Neck 2017;39(12):2381–2396; doi: 10.1002/hed.24895.
28. Makarenko V, Pavlychuk T, Kopchak A. Clinical manifestations of I-131 induced salivary gland dysfunction in patients with thyroid carcinoma. 2024.
29. Maruoka Y, Baba S, Isoda T, et al. A functional scoring system based on salivary gland scintigraphy for evaluating salivary gland dysfunction secondary to 131I therapy in patients with differentiated thyroid carcinoma. 2017.
30. Ming H, Yu H, Liu Y, et al. Effect of radioiodine therapy under thyroid hormone withdrawal on health-related quality of life in patients with differentiated thyroid cancer. Jpn J Clin Oncol 2022;52(10):1159–1166; doi: 10.1093/jjco/hyac113.
31. Missaoui AM. Hamza, F., Maaloul, M., Charfi, H., Ghrissi, W., Abid, M., Guermazi, F., Health-related quality of life in long-term differentiated thyroid cancer survivors: A crosssectional Tunisian-based study. 2022.
32. Nabaa B, Takahashi K, Sasaki T, et al. Assessment of salivary gland dysfunction after radioiodine therapy for thyroid carcinoma using non-contrast-enhanced CT: the significance of changes in volume and attenuation of the glands. AJNR Am J Neuroradiol 2012;33(10):1964–1970; doi: 10.3174/ajnr.A3063.
33. Rogers SN. Mepani, V., Jackson, S., Lowe, D., Health-related quality of life, fear of recurrence, and emotional distress in patients treated for thyroid cancer. British Journal of Oral and Maxillofacial Surgery 2017;55:666–673.
34. Selvakumar T, Nies M, Klein Hesselink MS, et al. Long-Term Effects of Radioiodine Treatment on Salivary Gland Function in Adult Survivors of Pediatric Differentiated Thyroid Carcinoma. The Journal of Nuclear Medicine 2019;60(2):172–177.
35. Solans R, Bosch JA, Galofré P, et al. Salivary and lacrimal gland dysfunction (Sicca syndrome) after radioiodine therapy. 2001.
36. Tabari A, Farrokh F, Bakhshi M, et al. Subjective and objective effects of radioiodine therapy on the sense of smell. Eur Arch Otorhinolaryngol 2024;281(10):5319–5324; doi: 10.1007/s00405-024-08761-3.
37. Walter MA, Turtschi CP, Schindler C, et al. The dental safety profile of high-dose radioiodine therapy for thyroid cancer: long-term results of a longitudinal cohort study. J Nucl Med 2007;48(10):1620–1625; doi: 10.2967/jnumed.107.042192.
38. Wu J, Feng H, Ouyang W, et al. Systematic evaluation of salivary gland damage following I-131 therapy in differentiated thyroid cancer patients by quantitative scintigraphy and clinical follow-up. Nucl Med Commun 2015;36(8):819–826; doi: 10.1097/MNM.0000000000000325.
